# Hsp70 (HSPA1) Lysine Methylation Status as a Potential Prognostic Factor in Metastatic High-Grade Serous Carcinoma

**DOI:** 10.1371/journal.pone.0140168

**Published:** 2015-10-08

**Authors:** Magnus E. Jakobsson, Anders Moen, Ben Davidson, Pål Ø. Falnes

**Affiliations:** 1 Department of Biosciences, University of Oslo, N-0316, Oslo, Norway; 2 Department of Pathology, Oslo University Hospital, Norwegian Radium Hospital, N-0424, Oslo, Norway; 3 University of Oslo, Faculty of Medicine, Institute of Clinical Medicine, N-0316, Oslo, Norway; Texas A&M University, UNITED STATES

## Abstract

Cellular proteins are subject to frequent methylation on lysine residues, introduced by specific methyltransferases, and each lysine residue can receive up to three methyl groups. Histone methylations, which are key determinants of chromatin state and transcriptional status, have been subject to particularly intense studies, but methylations on non-histone protein substrates are also abundant and biologically significant. Numerous studies have addressed lysine methylation in the realm of cancer biology. A recent study used an antibody-based approach to investigate the methylation of Lys-561 of the stress-inducible Hsp70 protein HSPA1, focusing exclusively on dimethylated HSPA1, concluding that it was elevated in cancer [Cho et al. (2012), *Nat*. *Commun*.,3, 1072]. In the present study, we have performed a more extensive analysis of HSPA1 methylation status in cancer samples, using protein mass spectrometry. We found that the four methylation states of Lys561 on HSPA1 (un-, mono-, di- and trimethylated) could be measured accurately and reproducibly in samples from carcinomas. We investigated HSPA1 methylation in 70 effusions, representing 53 high-grade serous ovarian carcinomas and 17 breast carcinomas. Notably, we found the trimethylated form of HSPA1 to be predominant in the cancer samples. HSPA1 methylation was studied for association with clinicopathologic parameters, including chemotherapy response and survival. The trimethylated form was more prevalent in breast carcinoma effusions (p = 0.014), whereas the dimethylated (p = 0.025), monomethylated (p = 0.004) and unmethylated (p = 0.021) forms were overrepresented in the ovarian carcinomas. For the ovarian carcinomas, the monomethylated (p = 0.028) and unmethylated (p = 0.007) forms were significantly related to the presence of higher residual disease volume, while the unmethylated form was significantly associated with poor overall (p = 0.015) and progression-free (p = 0.012) survival. In conclusion, lysine methylation of HSPA1 differs between metastatic breast and ovarian carcinoma, and unmethylated HSPA1 shows potential as a prognostic marker in high-grade serous carcinoma.

## Introduction

The altered properties of cancer cells, compared with normal cells, mainly result from perturbation of various cellular signaling pathways, mediated by mutation or altered expression of genes encoding signaling-associated proteins. A key component of cellular signaling is the post-translational modification of proteins, where specific enzymes mediate the attachment of small chemical groups, and in some cases larger moieties like peptides, onto cellular proteins. Post-translational modification can affect the function of a protein in various ways, *e*.*g*. by directly affecting its activity or stability, or by modulating its interaction with small molecular ligands or with macromolecules such as proteins, nucleic acids, sugars and lipids. Phosphorylation is arguably the most important and intensely studied post-translational modification, but a wealth of studies, mainly performed from year 2000 and onwards, have revealed a very important role also for protein methylation.

Proteins are mainly methylated on lysine and arginine residues, and these modifications are introduced by specific methyltransferases (MTases) [1,2]. Lysine methylation has been particularly intensively studied in the context of histone proteins, which are important components of chromatin. Lysine methylation occurs primarily on the flexible N-terminal tails that protrude from the otherwise globular histone proteins, and the methylation pattern on the histone tails is considered an important regulator of transcriptional activity and packing of chromatin [3]. Histone lysine methylation and protein phosphorylation share several notable features: i) the modifications can be reversed by specific enzymes, *i*.*e*. lysine demethylases and protein phosphatases, respectively; ii) so-called reader domains can specifically recognize the modified (or sometimes unmodified) residue; iii) genes that encode proteins responsible for introducing, recognizing or removing such modifications are frequently mutated or over-expressed in cancer, and have therefore attracted considerable attention as drug targets and diagnostic/prognostic markers.

Lysine methylations on histone proteins are introduced by specific lysine specific methyltransferases (KMTs), and each lysine residue can receive up to three methyl groups, thus generating four possible states (un-, mono-, di-, trimethylated; me0, me1, me2, me3). All but one of these enzymes, DOT1L, belong to a methyltransferase family that share a defining SET-domain [4]. DOT1L, on the other hand, is a member of a distinct enzyme family, the seven beta-strand (7BS) methyltransferase family [5,6]. The lysine methylation patterns on histones are often altered in cancer, and during recent years, considerable efforts have been put into developing specific inhibitors targeting DOT1L and members of the SET domain enzymes family [7,8]. Several of these have entered clinical trials and hold promise as future cancer drugs [7,8]. Similarly, specific inhibitors of lysine specific demethylases that remove methyl groups from lysines on histones have been developed [7,9].

In addition to the much-studied lysine methylations on histones, numerous other cellular proteins have been shown to be subject to lysine methylations, and recent studies have identified several novel human lysine methyltransferases that target non-histone substrates, many of which belong to the 7BS MTase family. We and others have discovered that the so-called MTase Family 16 (MTF16), a subfamily of the 7BS MTases, contain several novel human KMTs [10–15]. Recently, it was shown that one of the previously uncharacterized MTF16 enzymes, METTL21A, catalyzes methylation of a conserved lysine residue found in several human Hsp70 (HSPA) proteins, corresponding to Lys561 in the inducible Hsp70 protein HSPA1 [10,12]. Human HSPA1 protein has been strongly linked to cancer and is often upregulated in tumors, and increased levels correlate with poor prognosis [16,17]. Interestingly, a link between Hsp70 methylation in cancer was recently suggested, as dimethylation of Lys561 in HSPA1 appeared to be elevated in cancer samples, relative to normal tissue, using a HSPA1-K561me2 specific antibody [18]. However, this study did not investigate other HSPA1-K561 methylation states.

In the present study, we used mass spectrometry to measure the levels of all the four possible methylation states (me0, me1, me2, me3) for Lys561 in HSPA1 across a series of ovarian high-grade serous carcinomas (HGSC) and breast carcinomas. Our results show that HSPA1 methylation in small amounts of cancer specimen can be robustly measured. Moreover, the methylation levels differ in these two cancers, and are informative of outcome in HGSC.

## Materials and Methods

### Effusions

Specimens were submitted to the Department of Pathology at the Norwegian Radium Hospital during 1998–2004. A total of 70 effusions (53 HGSC, 17 breast carcinomas) were analyzed for HSPA1 methylation status. HGSC specimens (41 peritoneal, 12 pleural) were obtained from 53 patients diagnosed with primary tumor of the ovary (n = 45), peritoneum (n = 7) or the fallopian tube (n = 1). Twenty-seven effusions were obtained at diagnosis, prior to the administration of chemotherapy, whereas 26 effusions were obtained after chemotherapy, the majority at disease recurrence. Clinicopathologic data for HGSC patients are detailed in **Table 1**. Breast carcinoma effusions consisted of 17 effusions (14 pleural, 2 peritoneal, 1 pericardial) obtained from 16 patients (1 patient with 2 effusions) with histologically-verified infiltrating duct (n = 16) or lobular (n = 1) carcinoma of the breast. The majority of these specimens (n = 14) were obtained at disease recurrence.

**Table 1:** Clinicopathologic data of the HGSC effusion cohort (n = 53 patients)^a^.

FIGO stage			Residual disease			Chemoresponse after primary treatment^c^				
III	IV	NA^b^	≤1 cm	>1 cm	NA	CR	PR	SD	PD	NE
24	27	2	18	26	9	24	5	3	14	7

^a^The patients were aged 35–87 years (mean = 62) and displayed CA125 values of 11–43800 U/ml (mean = 4856) at diagnosis.

^b^NA = Non-available.

^c^CR = complete response, PR = partial response, SD = stable disease, PD = progressive disease, NE = disease response after chemotherapy could not be evaluated because of adverse effects, normalized CA-125 after primary surgery or missing CA-125 information and no residual tumor.

Effusions were centrifuged immediately after tapping and cell pellets were fresh-frozen at -70°C in equal amounts of RPMI medium containing 50% calf serum and 20% dimethylsulfoxide. Smears and H&E-stained cell block sections were reviewed by a surgical pathologist experienced in cytopathology (BD). Diagnoses were established based on morphology and immunohistochemistry. The relative amount of tumour cells in the cancer effusions was estimated microscopically by assessing cell morphology, as well as by immunohistochemistry, using antibodies directed against epithelial (carcinoma) and mesothelial cell epitopes, as previously described [19,20]. Only samples containing >50% of tumor cells were included in this study. The Regional Committee for Medical Ethics in Norway approved the study (Ethics approval S-04300) and granted dispensation from obtaining informed consent since the majority of patients were deceased at the time of application.

### Mass spectrometry analysis

Cells were lysed in ice-cold NP-40 lysis buffer (1% NP-40, 10% glycerol, 20 mM Tris-HCl pH 7.5, 137 mM NaCl, 1 mM sodium vanadate, 1 mM phenylmethyl sulphonyl fluoride (PMSF), 0.02 mg/ml each of aprotinin, leupeptin and pepstatin and 10μl/ml each of phosphatase inhibitor cocktail I and II). All protease inhibitors were purchased from Sigma Aldrich (St. Louis, MO). Lysates were sonicated, followed by centrifugation to collect the lysate supernatant.

Quantitative analysis of HSPA1-Lys561 was essentially performed as described before [12]. In brief, protein extracts from tumor effusions were separated by SDS-PAGE, following which the region surrounding the 70 kDa marker was excised and digested with the endoprotease Asp-N (Roche, Mannheim, Germany). Proteolytic peptides were thereafter separated and analyzed using a liquid chromatography (LC) coupled to tandem mass spectrometry (MS/MS) setup using collision induced fragmentation. In detail, peptide samples were concentrated using a Hypercarb 5μm particle column (G&T Septech AS) and separated with a C_18_ column (GlycproSIL C18-80Å, Glycpromass). Samples were washed with a mobile phase consisting of formic acid (0.1% (v/v)) and acetonitrile (2.5% (v/v)) and eluted with an increasing gradient acetonitrile. The LC setup was coupled to a LTQ Orbitrap XL mass spectrometer (Thermo Scientific) via nanoelectrospray.

Chromatograms representing the different methylated forms of Lys561 were generated by gating for mass-to-charge ratios corresponding to the four methylation states of Asp555–Ala565 of HSPA1 using Xcalibur 2.0 (Thermo Scientific, Waltham MA, USA). Selective ion setting used were m/z = 573.8037 (me0), 580.8115 (me1), 587.8193 (me2) and 594.8272 (me3) +/- 10 p.p.m. (z = 2). The relative abundance of the different methylated forms was determined by dividing the observed signal for the discrete forms, determined by integration using Xcalibur 2.0 (Thermo Scientific, Waltham MA, USA), by the sum of signals for all forms.

### Statistical analysis

Statistical analysis was performed applying SPSS (Version 21, Chicago, IL). Probability of <0.05 was regarded as statistically significant. The association between tumor type (HGSC vs. breast carcinoma) and HSPA1 methylation status in effusions was studied using the Mann-Whitney U test. The same test was applied to study the association between HSPA1 methylation status in HGSC effusions and clinicopathologic parameters. The latter were categorized as follows: Age: ≤60 vs. >60 years; effusion site: peritoneal vs. pleural; FIGO stage: III vs. IV; residual disease volume (RD): ≤1 cm vs. >1 cm; chemotherapy status: pre- vs. post-chemotherapy specimens; response to chemotherapy for primary disease and for disease recurrence: complete vs. partial response/stable disease/progression. The paired-sample T-test was used to analyze the association between HSPA1 methylation status and CA 125 levels.

Progression-free and overall survival (PFS; OS) were calculated from the date of the last chemotherapy treatment/diagnosis to the date of recurrence/death or last follow-up, respectively. Univariate survival analyses of PFS and overall survival (OS) were executed using the Kaplan-Meier method and log-rank test. Platinum resistance was defined as PFS≤6 months according to guidelines published by the Gynecologic Oncology Group [21] and progressive disease or recurrence was evaluated by the RECIST criteria [22]. Multivariate analysis was performed using the Cox Regression Model (Enter function).

## Results

### HSPA1 methylation status differs among ovarian and breast carcinomas

**Fig 1:**
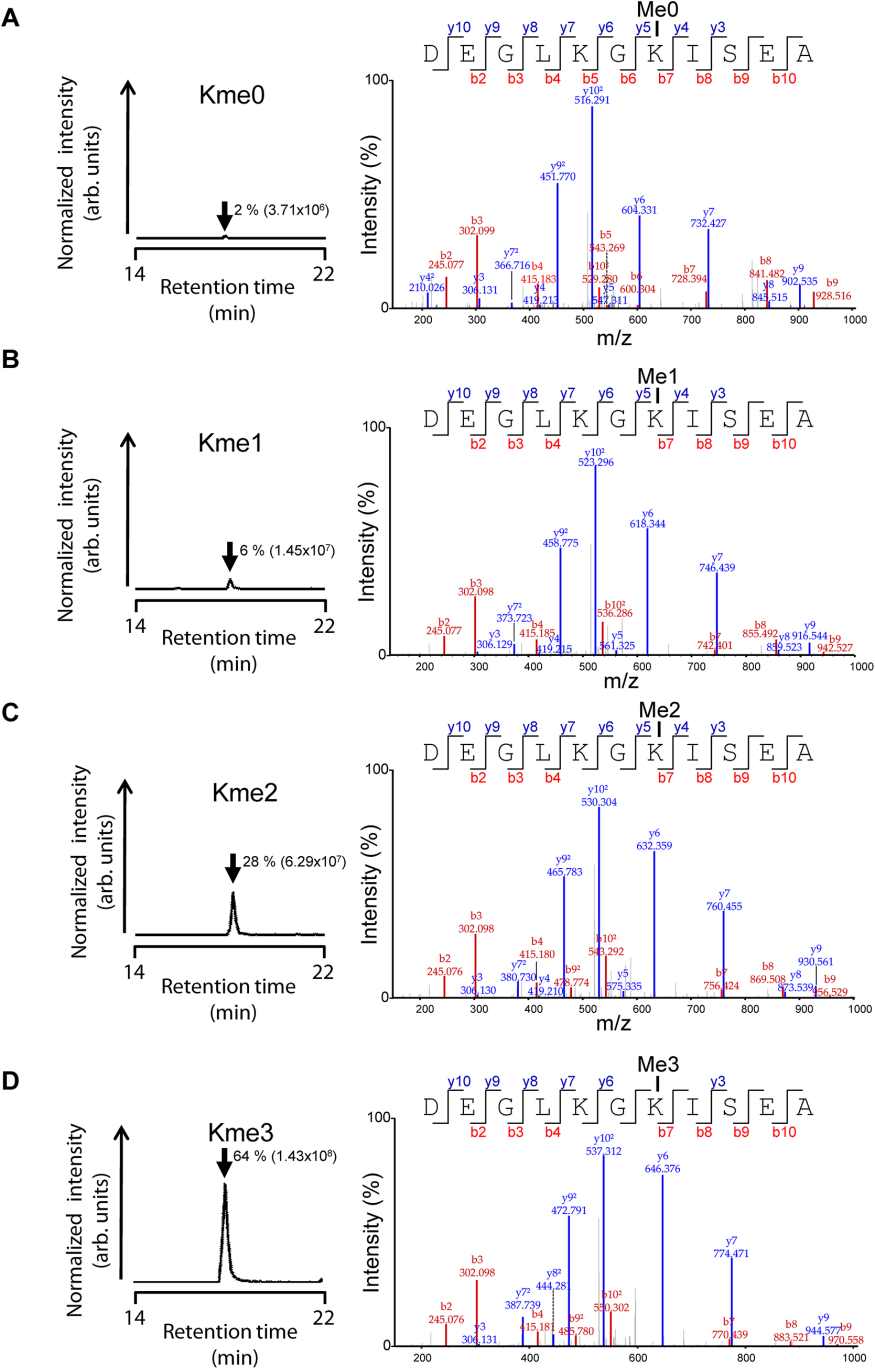
Representative mass spectrometry data from quantitative analysis of HSPA1-Lys561 methylation status. (A)-(D) Methylation status of Lys561 in HSPA1 in tumor sample # 14. Left panels, chromatograms were generated by gating for mass-to-charge ratios of the (A) unmethylated (me0), (B) monomethylated (me1), (C) dimethylated (me2) and (D) trimethylated (me3) state of the AspN-generated proteolytic peptide encompassing Asp555-Ala565 in HSPA1. The elution time (arrow) and the area under each curve (in brackets), as well as the calculated relative abundance (as percentage) of the various lysine methylation states, are indicated. Right panels, annotated tandem mass spectra supporting the identity of analyzed peptides.

We previously investigated the methylation status of HSPA1 in human cell lines, and to assess methylation in cancer samples, a similar approach was used [12]. In short, the four possible methylation states of the Lys561-containing Asp-N-generated peptide encompassing residues 555–565 in HSPA1 were analyzed by tandem mass spectrometry coupled to liquid chromatography (LC-MS/MS). From the resulting extracted ion chromatograms, the areas under the relevant peaks were used to calculate the relative amounts of the four methylation states (examples are shown in **Fig 1**), as well as the number of methyl groups on Lys-561 in HSPA1.

**Fig 2:**
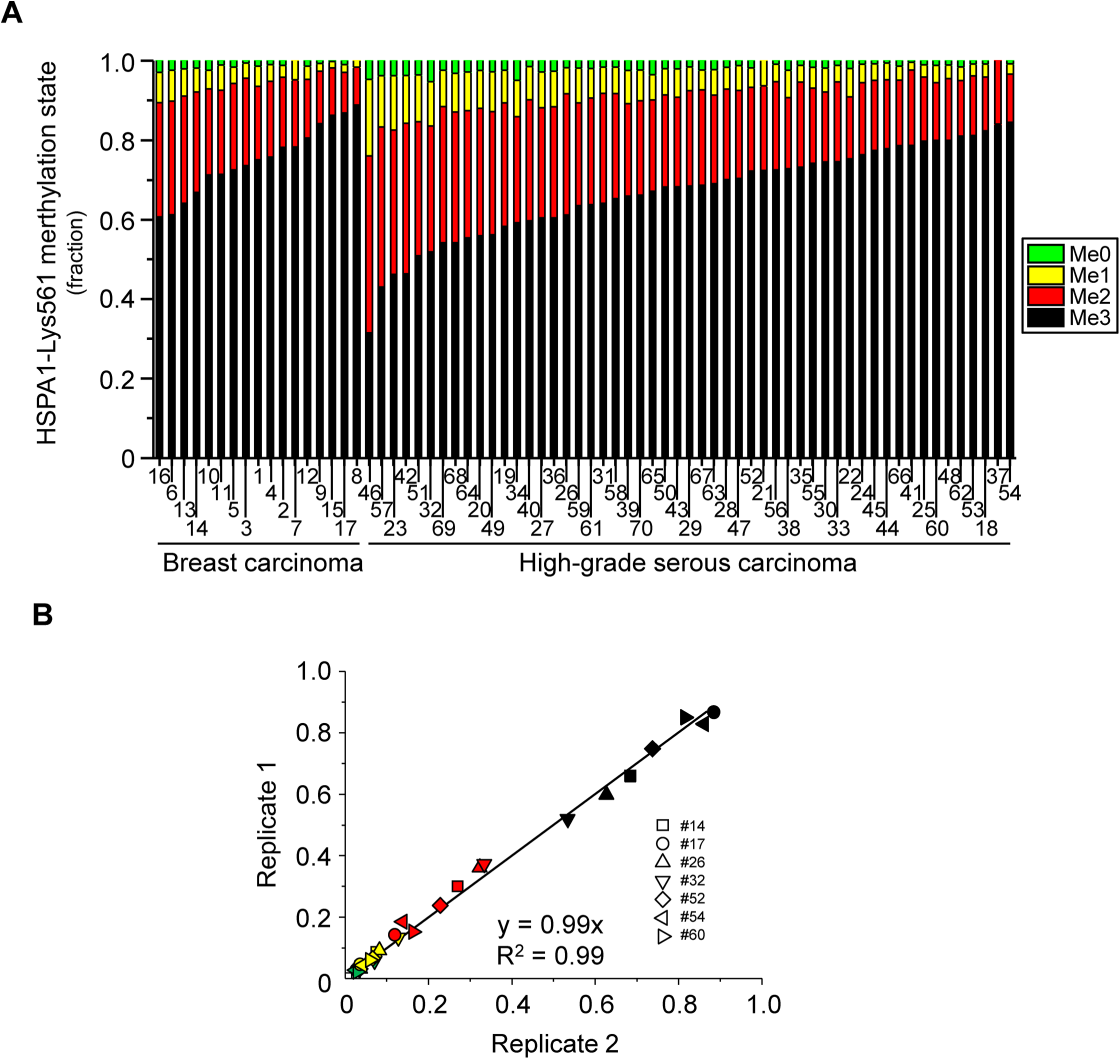
Assessment of HSPA1-Lys561 methylation status in HGSC and breast carcinoma effusions. (A) Quantitative mass spectrometry analysis of HSPA1-Lys561 methylation. Samples were analyzed as exemplified in Fig 1. The relative abundance of the various methylation states (me0, me1, me2 and me3) of Lys561 in HSPA1 in 17 breast carcinoma (cases # 1–17) and 53 HGSC (cases # 18–70) samples is shown. The data are grouped by cancer type and sorted by increasing relative abundance of me3. (B) Reproducibility of the analysis. A second analysis of 2 randomly chosen breast cancer samples and 5 randomly chosen HGSC samples was performed, and the results were plotted versus the original data set. Data points for the various lysine methylation states are color-coded as in (A) and each tumor sample is represented by a unique geometrical figure.

In the present study we have analysed 70 cancer effusions, 53 from HGSC and 17 from breast carcinoma. These samples were selected due to the availability of sufficient material for the LC-MS/MS analysis, as well as the availability of clinicopathological information for the corresponding cases. Analysis of the 70 effusions showed substantial differences in the relative amounts of the four different methylation states varied substantially across samples (**Fig 2A**). In particular, the monomethylated and dimethylated states, which where detectable in all samples, showed a variation of ~13- and ~5-fold, respectively, across the tumor samples. Using the Mann-Whitney U test for statistical analysis, we observed significantly higher HSPA1 methylation levels in breast carcinoma relative to HGSC. Total HSPA Lys-561 methylation (methyl groups per molecule, p = 0.010), as well as the trimethylated form (me3; p = 0.014) were elevated in breast carcinoma effusions, whereas, correspondingly, the lower methylation states dimethyl (me2; p = 0.025), monomethyl (me0, p = 0.004) and no methylation (me0, p = 0.021) were more abundant in HGSC. Due to the limited amounts of patient material available, as well as the time-consuming nature of the analyses (each sample requires an LC-MS/MS run of 65 minutes duration), samples were only analyzed once. However, since all four methylation states were measured simultaneously in a single LC-MS/MS run, error sources such as run-to-run variation and variation in sample loading were eliminated, and the measurements of the relative levels of the four methylation states may be regarded as quite accurate. Nevertheless, to obtain an estimate of the experimental error associated with the measurements, a subset of the samples was analyzed twice. The duplicate measurements of the same sample gave nearly identical results (**Fig 2B**), demonstrating that the technical reproducibility of the analysis is indeed very good, and that only minor experimental error is likely to be associated with the data obtained by single analysis of the tumor specimens.

### HSPA1 methylation status in HGSC is associated with clinicopathologic parameters and survival

We also performed a correlative analysis of HSPA1 methylation status and various clinicopathological parameters. Since the majority of the cancer samples analysed for HSPA1 methylation were from HGSC, we focused the analysis on this subset of the samples. We found that significantly higher levels of trimethylated HSPA1 were found in specimens from patients diagnosed with FIGO stage IV disease compared to those with stage III (predominantly IIIC) disease (p = 0.011), whereas the opposite was true for the dimethylated (p = 0.015), monomethylated (p = 0.015) and unmethylated (p = 0.03) forms. Furthermore, the abundance of the monomethylated (p = 0.028) and unmethylated (p = 0.007) forms was increased in effusions from patients with a higher residual disease volume (RD), with no significant difference for the trimethylated and dimethylated forms. HSPA1 methylation status was unrelated to effusion site, previous chemotherapy, patient age, CA 125 (Cancer Antigen 125; serum biomarker for ovarian carcinoma) level at diagnosis, chemotherapy response at diagnosis or primary (intrinsic) chemoresistance in this cohort (p>0.05).

**Fig 3:**
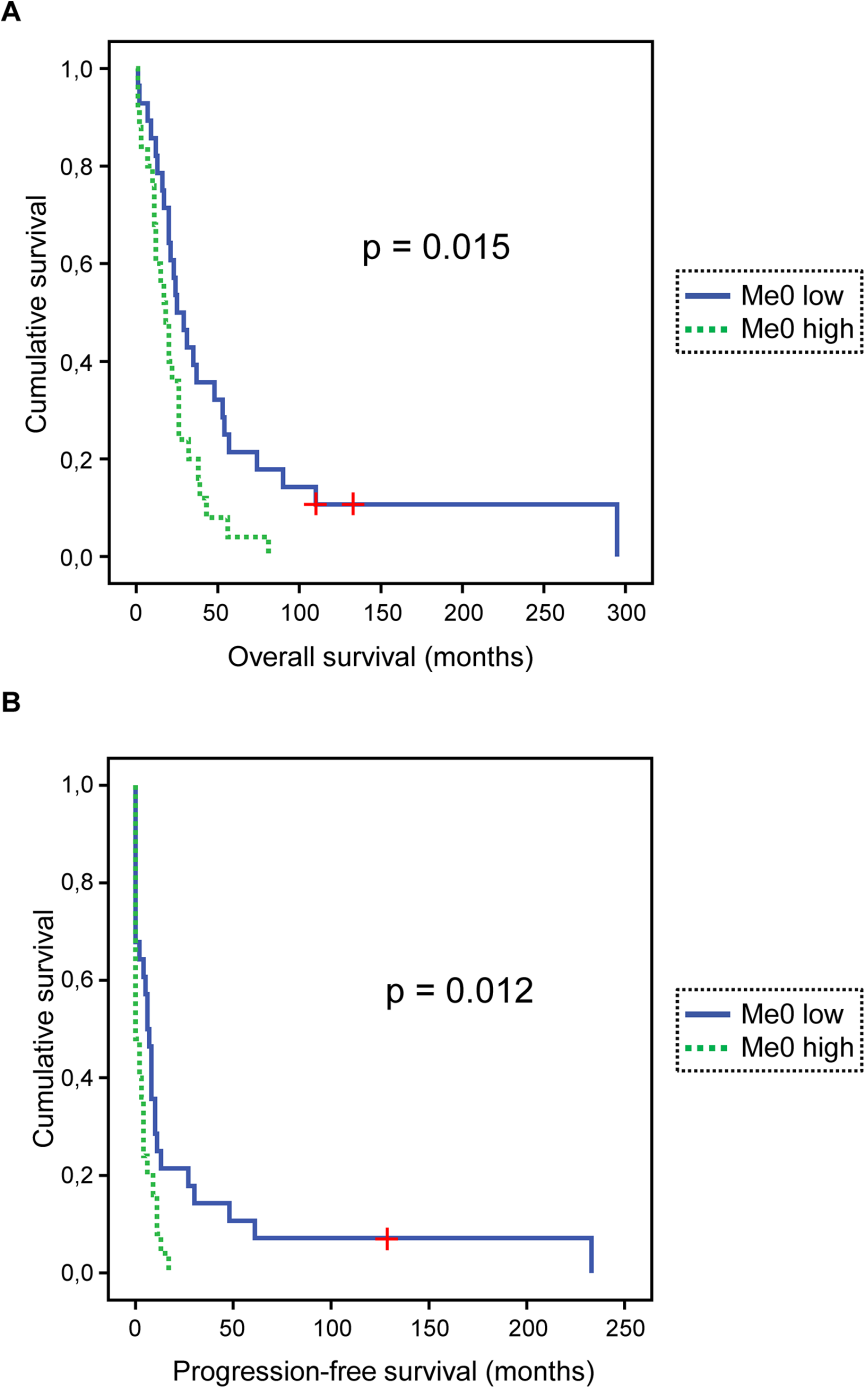
HSPA1 methylation status is associated with survival in HGSC. (A) Kaplan-Meier survival curve showing the association between levels of the unmethylated form of Lys561 on HSPA1 in effusions and overall survival (OS) for 53 HGSC patients. Patients with effusions with higher-than-median relative abundance of me0 (Me0 high; n = 25, dashed green line) had a mean OS of 22 months vs. 61 months for patients whose effusions showed lower-than-or-equal-to-median relative abundance of me0 (Me0 low; n = 28, solid blue line; p = 0.015). “+” indicates patients who were alive at the end of their follow-up period, and therefore were censored. (B) Kaplan-Meier survival curve showing the association between levels of the unmethylated form of Lys561 on HSPA1 in effusions and progression-free survival (PFS) for 53 HGSC patients. Patients with effusions with higher-than-median relative abundance of me0 (Me0 high; n = 25, dashed green line) had a mean PFS of 3 months vs. 26 months for patients whose effusions showed lower-than-or-equal-to-median relative abundance of me0 (Me0 low; n = 28, solid blue line; p = 0.012). “+” indicates one patient who was alive without signs of disease at the end of the follow-up period, and was, therefore, censored.

Survival data were available for all 53 patients with HGSC. OS (overall survival) ranged from 1 to 295 months (mean = 36 months, median = 22 months). PFS (progression-free survival) ranged from 0 to 233 months (mean = 13 months, median = 4 months). At the last follow-up, 1 patient was alive with no evidence of disease, 1 was alive with disease and 51 were dead of disease. Notably, we have previously analysed these cancer samples with respect to HSPA1 expression levels [23], but we did not find any association between HSPA expression and methylation. In univariate survival analysis, unmethylated HSPA1 was associated with poor OS (p = 0.015; **Fig 3A**) and PFS (p = 0.012; **Fig 3B**). Although an interesting association between unmethylated HSPA1 and survival was observed, no obvious correlation between overall (me1+me2+me3) HSPA1 methylation and survival appears to exist (S1 Fig).

The data underlying the results in Fig 3 are shown in Table 2. Among the clinical parameters, only RD volume was significantly related to outcome (p = 0.003 and p = 0.001 for OS and PFS, respectively). In summary, the various methylation states of HSPA1 correlate with clinical parameters, with low HSPA1 methylation being an indicator of poor outcome.

**Table 2:** HSPA1 methylation and patient survival in HGSC.

Patient #	HSPA 1 methylation state (%)				Me per HSPA1^a^	PFS (mo)	OS (mo)
	Me0	Me1	Me2	Me3			
18	0.7	3.3	13.6	82.4	2.78	0	31
19	2.3	8.3	31.1	58.3	2.45	0	18
20	2.4	9.5	32.1	56	2.42	0	81
21	0	6.2	21.4	72.4	2.66	13	57
22	1.9	7.1	15.6	75.3	2.64	17	39
23	3.7	13.7	36.3	46.2	2.25	4	20
24	0.8	4.7	18.1	76.4	2.70	0	13
25	0.9	3.1	16.2	79.8	2.75	6	25
26	1.7	6.6	30.6	61.2	2.51	0	7
27	2.8	8.9	27.8	60.4	2.46	9	56
28	1.6	5.6	22.7	70.2	2.62	10	48
29	1.5	6	24.1	68.5	2.60	7	20
30	1.8	6	17.7	74.5	2.65	11	29
31	1.5	6.6	27.8	64.1	2.55	48	110
32	5.2	11.1	31.7	51.9	2.30	3	20
33	1.1	4.2	20.1	74.7	2.69	8	37
34	4.9	9.2	26.7	59.3	2.41	13	38
35	1.1	4.2	21.5	73.2	2.67	0	12
36	2.6	8.9	28	60.5	2.46	0	3
37	0	0	15.9	84.1	2.84	30	54
38	2.4	6.9	17.8	72.9	2.61	0	11
39	2.4	8.3	23.2	66	2.53	11	43
40	1.4	8.3	30.5	59.7	2.48	6	21
41	0.3	2	19	78.8	2.76	10	53
42	3.6	12.1	37.9	46.4	2.27	0	17
43	2	7.1	22.6	68.3	2.57	0	26
44	0.5	4.2	17.4	77.9	2.73	27	90
45	0.7	4.1	17.7	77.5	2.72	8	74
46	4.6	19.3	44.5	31.6	2.03	11	32
47	1.2	6.3	22.2	70.4	2.62	5	24
48	1.1	3.3	15.6	80	2.75	8	35
49	2.8	10	31	56.2	2.41	0	7
50	2	6.6	23.3	68.2	2.58	0	15
51	3.5	11.8	33.7	50.9	2.32	4	22
52	1.7	5	21.1	72.2	2.64	0	2
53	0.7	3.1	15	81.2	2.77	0	17
54	0.7	2.6	12.1	84.6	2.81	0	1
55^b^	1.6	5.3	18.9	74.3	2.66	61	110
56	0.8	4.4	22.2	72.6	2.67	0	20
57	3.8	12.9	40.3	43.1	2.23	6	26
58^c^	1.5	6.7	26.4	65.4	2.56	127	132
59	1.8	8.7	25.9	63.6	2.51	4	23
60	1.1	4.3	14.6	80	2.74	0	16
61	1.9	7.4	26.8	63.8	2.52	0	1
62	1.5	3.5	14	81.1	2.75	2	9
63	2.2	6.4	22.3	69.1	2.58	0	2
64	2.8	9.6	32.1	55.5	2.40	2	11
65	3.4	6.5	22.9	67.2	2.54	0	10
66	1.3	3.5	16.5	78.7	2.73	233	295
67	2.3	5	24	68.7	2.59	0	12
68	3.1	9.7	32.9	54.2	2.38	0	1
69	2.4	9.1	34.3	54.2	2.40	2	26
70	2.3	7.8	23.7	66.3	2.54	4	12

^a^Number of methyl groups on Lys-561 in HSPA1, calculated from the data in the three previous columns according to the following formula: (Me1 + 2∙Me2 + 3∙Me3)/100

^b^Patient was alive with disease at the end of the follow-up period (110 mo), and was therefore censored in OS curve (Fig 3A)

^c^Patient was alive without disease after the end of the follow-up period (132 mo for OS and 127 mo for PFS), and was therefore censored in OS and PFS curves (Fig 3)

## Discussion

In the present study we used mass spectrometry to investigate the methylation status of the inducible Hsp70 protein HSPA1 in cancer, and found that HSPA1 methylation could be robustly and reproducibly measured across a panel of ovarian and breast carcinoma effusions. Moreover, we found that HSPA1 methylation showed associations with several clinical parameters, the most interesting one being that a high relative abundance of unmethylated HSPA1 was associated with poor prognosis.

Most studies on lysine methylation and cancer have focused on histone proteins. However, lysine methylation has also been shown to be essential for regulating cancer-relevant functions of nuclear non-histone proteins, such as the tumor suppressors Rb and p53 [24,25]. More recently, it was demonstrated that methylation of MAP3K2 by the cytosolic KMT SMYD3 promotes tumour formation through activation of the Ras-signalling pathway, establishing a role also for cytosolic lysine methylation in cancer [26]. As HSPA1 is predominantly a cytosolic protein, the present study lends further support to the notion that cytosolic lysine methylation is cancer-relevant.

Numerous reports have investigated HSPA1 expression in various cancers, and, generally, increased HSPA1 expression has been associated with poor prognosis and increased tumor grade [16,17]. The HGSC effusions that were investigated for HSPA1 methylation in the present study are part of a series which was previously analyzed for HSPA1 (HSP70) expression by immunohistochemistry, in which increased cytoplasmic HSPA1 expression was found to correlate with poor OS [23]. The high HSPA1 levels observed in particularly aggressive cancers are thought to reflect a strong requirement for protein chaperone function in rapidly growing cells with a high protein synthesis rate that also, due to genomic alterations, express increased amounts of aberrant proteins. However, we did not observe any correlation between HSPA1 methylation and expression for the HGSC samples, indicating that alterations in the degree of HSPA1 methylation are not secondary effects of changes in overall HSPA1 levels. Our observation that the various methylation states of HSPA1 are linked to clinicopathologic parameters can be explained by two alternative, but not necessarily mutually exclusive, models. First, the methylation status of Lys561 may be a determinant of the functional state of HSPA1 by altering the stability, interactome or subcellular localization of the protein. Alternatively, the methylation state of the residue may be a reflection of the abundance and turnover of the responsible methyltransferase, as well as the levels of the methyl donor, *S*- adenosylmethionine, which in turn is linked to metabolism. Actually, a role for methylation of Lys561 in HSPA1 in cancer has already been suggested. Cho *et al*. performed immunohistochemistry with an antibody generated to specifically recognize dimethylated HSPA1-K561, and observed increased signals in various tumors relative to normal tissue [18]. However, the authors also concluded, without substantial experimental evidence, that the bulk of cellular HSPA1 is unmethylated, whereas the dimethylated species is found specifically in the nucleus. This is clearly at odds with our results, as we found that only a minor fraction of HSPA1 is unmethylated, and that trimethylation is the predominant methylation state.

In conclusion, our results link the methylation status of HSPA1-Lys561 to cancer outcome, suggesting that methylation of this residue can be used as a diagnostic and/or prognostic marker. Previous studies have reported an elevated level of HSPA1 in serum as a biomarker for various cancers [27–29]. Our results strongly indicate that also assessing the HSPA1 methylation status of Lys561 would provide additional information, likely to increase the overall performance of HSPA1 as a biomarker. Finally, the observed correlation between HSPA1 methylation status and clinicopathologic parameters is interesting, but further research is required to elucidate a possible causative effect of HSPA1 methylation status on tumor formation and development. Such follow-up studies may for example investigate tumorigenesis in animal models deficient in METTL21A, the enzyme responsible for HSPA1 methylation.

## Supporting Information

S1 Fig Total methylation of Lys-561 in HSPA1 plotted versus overall (A) and progression-free (B) survival of HGSC patients. The curves were generated based on the data in Table 2.
(PDF)

## Abbreviations

7BS: seven beta strand
HGSC: high grade serous carcinoma
KMT: lysine specific methyltransferase
LC: liquid chromatography
MS/MS: tandem mass spectrometry
MTase: methyltransferase
MTF16: methyltransferase family 16
OS: overall survival
PFS: progression free survival
RD: residual disease volume
