# Supplementary figures and images for: Hsp70 (HSPA1) Lysine Methylation Status as a Potential Prognostic Factor in Metastatic High-Grade Serous Carcinoma
Source: PLoS One. 2015 Oct 8;10(10):e0140168. doi: 10.1371/journal.pone.0140168 (PMC4598032; doi:10.1371/journal.pone.0140168)

A

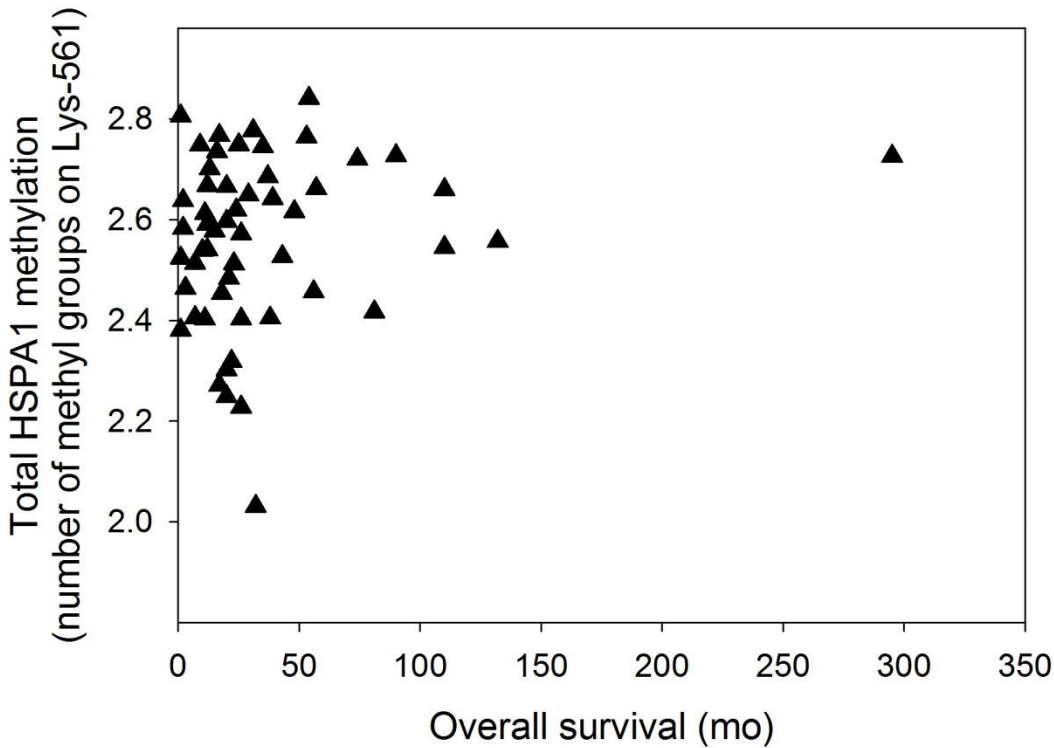

B

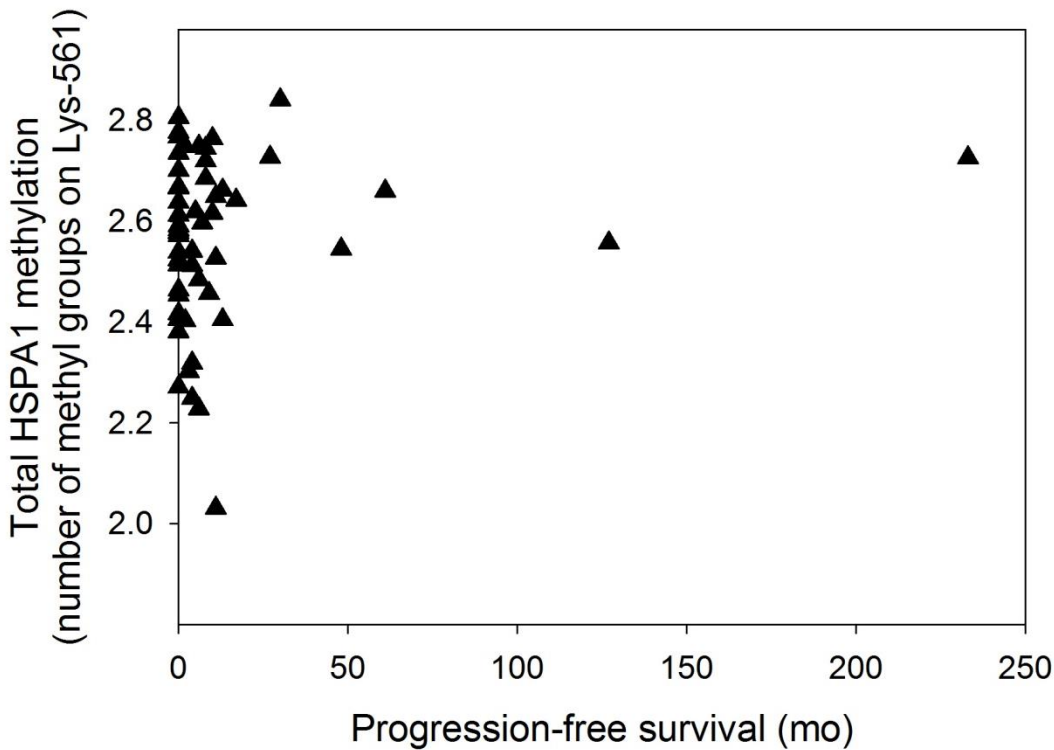

Supplement: S1 Fig — The curves were generated based on the data in Table 2. (PDF) [file pone.0140168.s001.pdf]
